# Deep learning based diagnosis for cysts and tumors of jaw with massive healthy samples

**DOI:** 10.1038/s41598-022-05913-5

**Published:** 2022-02-03

**Authors:** Dan Yu, Jiacong Hu, Zunlei Feng, Mingli Song, Huiyong Zhu

**Affiliations:** 1 Department of Oral and Maxillofacial Surgery, The First Affiliated Hospital, Zhejiang University School of Medicine, 79# Qingchun Road, Hangzhou, 310003 People’s Republic of China; 2 Computer Science and Technology, Zhejiang University, 38# Zheda Road, Hangzhou, 310027 People’s Republic of China

**Keywords:** Mathematics and computing, Dentistry, Medical imaging, Machine learning

## Abstract

We aimed to develop an explainable and reliable method to diagnose cysts and tumors of the jaw with massive panoramic radiographs of healthy peoples based on deep learning, since collecting and labeling massive lesion samples are time-consuming, and existing deep learning-based methods lack explainability. Based on the collected 872 lesion samples and 10,000 healthy samples, a two-branch network was proposed for classifying the cysts and tumors of the jaw. The two-branch network is firstly pretrained on massive panoramic radiographs of healthy peoples, then is trained for classifying the sample categories and segmenting the lesion area. Totally, 200 healthy samples and 87 lesion samples were included in the testing stage. The average accuracy, precision, sensitivity, specificity, and F1 score of classification are 88.72%, 65.81%, 66.56%, 92.66%, and 66.14%, respectively. The average accuracy, precision, sensitivity, specificity, and F1 score of classification will reach 90.66%, 85.23%, 84.27%, 93.50%, and 84.74%, if only classifying the lesion samples and healthy samples. The proposed method showed encouraging performance in the diagnosis of cysts and tumors of the jaw. The classified categories and segmented lesion areas serve as the diagnostic basis for further diagnosis, which provides a reliable tool for diagnosing jaw tumors and cysts.

## Introduction

Odontogenic cysts and tumors of the jaw are the second most common disease after tooth impaction in the oral and maxillofacial areas. The cysts and tumors of the jaw are usually painless and asymptomatic unless they grow so large as to involve the entire jawbone, causing noticeable swelling or weakening it to cause pathologic fractures^1,2^. Those manifested symptoms pose a severe threat to patient life quality. The majority of these cyst and tumor lesions can be identified at an earlier stage through a routine radiographic exam called the panoramic radiograph or orthopantomogram^3^. The treatment modalities for different types of cysts and tumors are different. Keratocystic odontogenic tumors (KCOTs), like other cystic lesions, are usually enucleated without radical jaw segmentation. Ameloblastomas (ABs), on the other hand, require more radical surgical removal than KCOTs, which drastically affects patients’ lives, causing facial deformity and subsequent social and emotional incompetence^4,5^.

Accurate diagnosis of different types of cysts and tumors is a challenging task. Some cysts and tumors have very similar radiological characteristics. In the orofacial region, accurate differentiation between various cystic lesions and ABs can be challenging. This is mainly due to their common presentations on conventional dental panoramic radiographs^6^. The main differentiating feature in diagnosing KCOT from ABs is the significant anteroposterior extension of the unilocular radiolucent lesion in the posterior mandible marrow space. KCOTs can sometimes also present as multilocular lesions, which further complicate its differentiation from ameloblastomas^4^. Misdiagnosis between these two lesions is therefore a common clinical pitfall. So, an auxiliary diagnostic method for cysts and tumors is significant.

Recently, deep learning approaches have achieved promising results in the medical image analysis area^7^. Inspired by the successful application of deep learning, several works^8–21^ adopted Convolutional Neural Network (CNN) to diagnose radiolucent lesions in the oral and maxillofacial area. Lee et al.^11^ adopted deep convolutional neural networks to screen orthognathic surgery, where the Grad-CAM^22^ is adopted to visualize whether the deep learning AI model considered and evaluated the correct region. However, the performance of deep learning-based methods heavily relies on a large number of labeled datasets. Existing CNN-based methods^18,23,24^ first pre-train the whole classification network on ImageNet^25^, and then finetune the network on several hundreds of lesion samples. The domain difference between normal image dataset ImageNet^25^ and medical radiographs is huge, which heavily reduces the robustness and performance of the above transfer learning-based methods^18,23^. On the other hand, the explainability of auxiliary diagnostic methods is an essential factor for diagnosing cysts and tumors. However, existing deep learning-based methods lack explainability, which is a disadvantage for diagnosing cysts and tumors. Furthermore, sufficient labeled samples can effectively improve the performance of deep learning-based methods. However, collecting and labeling massive lesion samples are time-consuming and heavily relies on the professional doctor’s experience. On the contrary, collecting massive healthy panoramic radiographs is more accessible and does not require a professional doctor’s annotation.

Therefore, the aim of this study is to develop an explainable and reliable method to diagnose cysts and tumors of the jaw with massive panoramic radiographs of healthy people based on deep learning. We develop a two-branch framework for diagnosing cysts and tumors of the jaw, where the position consistency constraint between the segmentation results and the response maps of classification is adopted to improve the reliability and explainability of the predicted results. Experiments show that the proposed two-branch network can simultaneously predict the category and area of lesion samples, which can serve as the diagnostic reference for further diagnosis of doctors.

## Related works

Recently, the deep learning technique has achieved promising results in tumor image analysis tasks^12^, such as brain tumor image analysis^13^, breast tumor analysis^14^, and liver tumor analysis^15^. Inspired by the successful application of deep learning techniques, several works are proposed to diagnose radiolucent lesions in the oral and maxillofacial area, which can be divided into classification methods and detection methods.

For the former category, Poedjiastoeti et al.^23^ adopted the VGG-16 network for classifying the ameloblastomas and KCOTs. The VGG-16 network is pretrained on the ImageNet dataset and finetuned with 400 image samples. Lee et al.^18^ adopted the pretrained GoogLeNet Inception-v3 architecture to classify odontogenic keratocysts, dentigerous cysts, and periapical cysts with 1140 panoramic and 986 CBCT images. For the latter category, Ariji et al.^16^ proposed the first object detection framework (a pre-trained fully convolutional network) to detect the lesion area and classify them. Kwon et al.^17^ developed a deep CNN modified from YOLOv3 for detecting and classifying odontogenic cysts and tumors of the jaw with 1282 panoramic radiographs. Yang et al.^26^ adopted the YOLOv2 for detecting and classifying dentigerous cyst, odontogenic keratocyst (OKC), and ameloblastoma with 1603 panoramic radiograph samples.

However, due to limited lesion samples, most above deep networks are firstly pretrained on other datasets such as ImageNet, then are finetuned with the jaw panoramic radiographs. The domain differences have severe limitations on the robustness and performance of those pre-trained networks. What’s more, deep learning based diagnosis methods for cysts and tumors of the jaw have a potential deficiency noninterpretability, which severely constrains the application of existing deep learning based methods. In several works^11,23^, the Grad-CAM^22^ technique is adopted to visualize the category-related areas, the reliability of which will be disturbed by the inaccurate prediction.

## Materials and methods

### Dataset

**Figure 1:**
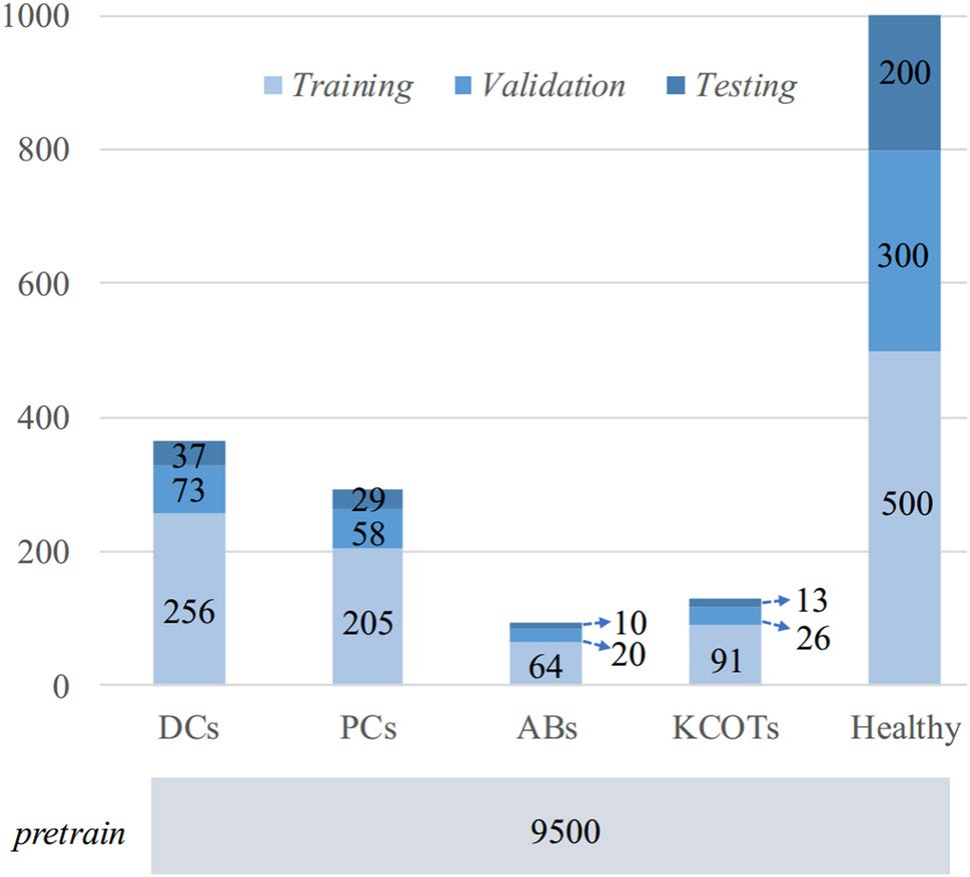
The dataset statistic of panoramic radiographs. KCOTs: Keratocystic odontogenic tumors; Abs: Ameloblastomas; DC: dentigerous cysts; PC: periapical cysts. In our experiment, healthy panoramic radiographs are split into 9500 samples for pretraining and training, 300 samples for validation, and 200 samples for testing. For lesion samples, 70%, 20%, and 10% samples are used for training, validation, and testing.

This study was conducted at the First Affiliated Hospital, Zhejiang University School of Medicine. Waiver of informed consent for data collection was approved by the Clinical Research Ethics Committee of the First Affiliated Hospital, Zhejiang University School of Medicine (IIT20200430A-R2). Since 2005, the World Health Organization (WHO) has labeled OKCs as keratocystic odontogenic tumors (KCOTs) and has classified OKCs as tumors according to their behavior. Based on histopathological examinations by a board-certified oral pathologist at First Affiliated Hospital, Zhejiang University School of Medicine, we collected 10,000 panoramic radiographs of healthy peoples and 872 lesion samples, which contains 356 dentigerous cysts (DC) samples, 292 periapical cysts (PC) samples, and 94 ameloblastoma (AB) samples, 130 keratocystic odontogenic tumor (KCOT) samples. Those samples were acquired between December 2018 and February 2020. Even if histologically confirmed, all difficult to distinguish cases because of the severe distortion, artificial noise, blur, and poor quality in the radiographic image were excluded. For each lesion sample, an experimental dentist annotates the lesion area mask and lesion category. In our experiment, healthy panoramic radiographs are split into 9500 samples for pretraining and training, 300 samples for validation, and 200 samples for testing. For lesion samples, 70%, 20%, and 10% samples are used for training, validation, and testing. Figure 1 summarizes more details about the collected dataset.

### Image preprocessing and augmentation

The size of the original panoramic radiograph is about 3000 × 1500, which is too large for the normal deep network. What’s more, through statistical calculation of lesion area position, we find that peripheral areas don’t contain lesions. So, we get the common center areas by throwing away useless peripheral areas, which can maintain lesion-related patches and remove useless parts as much as possible. In the experiment, the cropped patches are resized into 512 × 256. The data augmentation strategies we adopted include horizontal flipping, cut-and-pasting, and patch-covering based on the characteristics of medical images. The cut-and-pasting strategy denotes cutting the lesion area and pasting it on a healthy sample. The patch-covering denotes covering the lesion area and healthy area with a gray patch of lesion samples to augment lesion samples’ diversity. We survey lesion area size on all lesion samples, which gives the minimal and maximal size of the lesion area. For the lesion area of a lesion sample, the cover-patch size is randomly generated between the size of the lesion area and the maximal size. For the healthy area of a lesion sample, the cover-patch size is randomly generated between the minimal and the maximal sizes. In our experiment, a lesion sample will be covered with 20 patches, where half the patches are generated for covering the lesion area and the rest half the patches are used for covering the healthy area of the lesion sample. It’s worth noting that the patch-covering can generate very similar samples for the same lesion sample, which is an advantage for enhancing the reliability of predicted output’s interpretability.

### Model architecture

The performance of deep learning-based methods highly relies on the number of training samples. The human can observe abnormity through mass observation of massive healthy samples. Inspired by the above fact, we propose a deep learning-based diagnosis method for cysts and tumors of the jaw with massive healthy samples. The proposed framework is composed of two parts: a self-supervised network and a two-branch network. The self-supervised network is adopted to learn basic knowledge from massive healthy samples. Then, the knowledge of the self-supervised network is used in the two-branch network by replacing the encoder of the two-branch network with the pretrained encoder of the self-supervised network.

For improving the reliability and explainability of diagnosis results, the two-branch network is devised to be composed of a classification sub-branch and segmentation sub-branch. The segmentation sub-branch will predict the lesion area, which can serve as the diagnosis reference for dentists and oral surgeons to diagnose the jaw tumors and cysts further.

**Figure 2:**
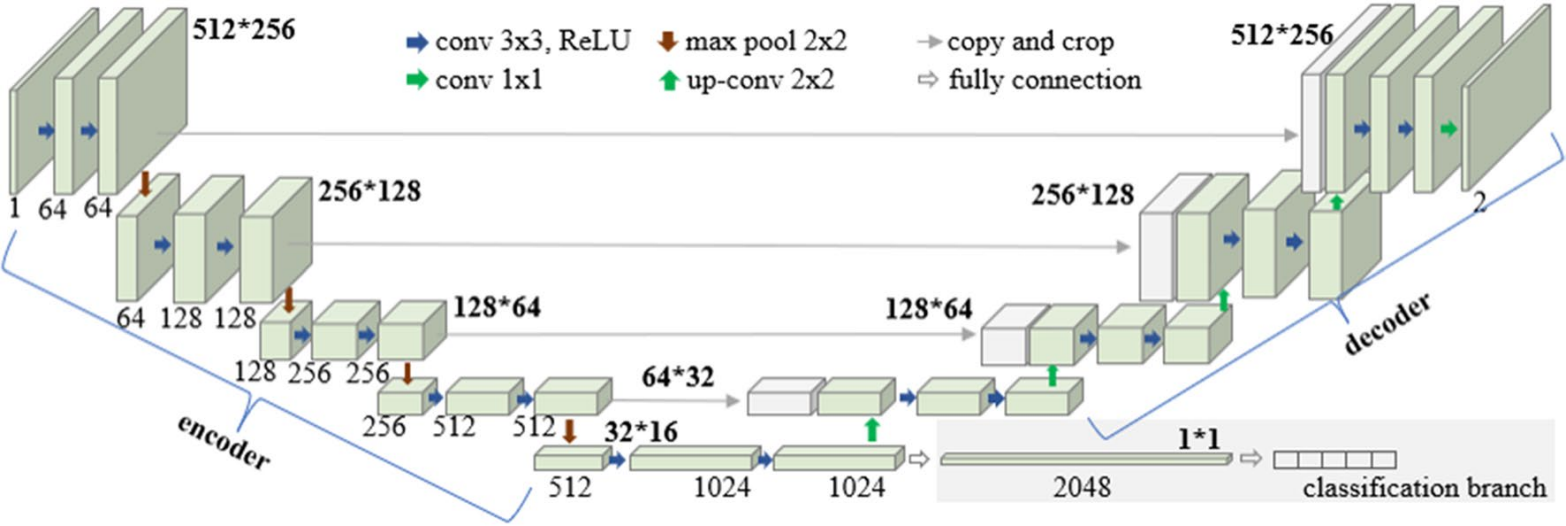
The two-branch network architecture.

In the experiment, the self-supervised network we adopted is MoCoV2^27^. We adopted Unet^28^ as the segmentation sub-branch. The classification sub-network, the self-supervised network, and the segmentation sub-branch share the same encoder. The remaining part of the classification sub-branch contains an average pooling layer, 2048 fully connection layers. The two-branch network architecture is given in Fig. 2.

### Model training and inference

In the experiment, the whole model is trained in two stages. The self-supervised network is firstly trained on 9500 healthy samples with the default parameter setting in the work of Chen et al.^27^. Then, the pre-trained encoder is used to initialize the encoder of the two-branch network. Next, the classification sub-branch and segmentation sub-branch are trained with CrossEntropy loss ${L}_{CE}$ and Mean Squared Error on 872 lesion samples and 500 healthy samples as follows:

$${L}_{CE}=\frac{1}{K}\sum_{k=1}^{K}{y}_{k}\;{\text{log}}\;{p}_{k},$$

$${L}_{MSE}=\frac{1}{K}\sum_{k=1}^{K}{\Vert \overline{{M }_{k}}-{M}_{k}\Vert }_{2}^{2},$$

where, $K$ is the number of training samples, ${y}_{k}$ and ${p}_{k}$ denote the ground-truth and predicted probability of $k$-th sample, ${M}_{k}$ and $\overline{{M }_{k}}$ denote the ground-truth and predicted mask of the $k$-th sample. For the classification and segmentation sub-branches, the learning rates are ${1e}^{-3}$ and ${1e}^{-2}$, respectively. In the training stage, the weights for the classification loss ${L}_{CE}$ and segmentation loss ${L}_{MSE}$ are set to 1:1. 872 lesion samples are composed of 648 cyst samples (DCs: 356, PCs:292) and 224 tumor samples (ABs: 94, KCOTs: 130). Healthy samples used in the training stage are also used in the pretrain stage.

To improve the reliability of the predicted results, we adopted the annotated segmentation mask to constrain the consistency between the segmentation results and classification results. Grad-CAM^22^ can visualize the high response to the final predicted probability. For the lesion samples, the feature of the lesion area should have a major contribution to the final classification, while the healthy areas should have no contribution. So, we adopted the annotated lesion mask to constrain the lesion area and healthy area with high gradient responses and no gradient responses regarding the final classification label. The constrain ${L}_{constrain}$ is implemented by maximizing the responses around the lesion areas and minimizing the responses in the unrelated background area as follows:

$${L}_{constrain}=\frac{1}{K}\sum_{k=1}^{K}\left(\sum_{n=1}^{N}(1-\overline{{M }_{k}^{d}}\left[n\right])*{R}_{k}\left[n\right]-\sum_{n=1}^{N}\overline{{M }_{k}^{d}}\left[n\right]*{R}_{k}\left[n\right]\right),$$

where, $N$ is the multiplication of width and height of the last layer feature map, ${R}_{k}\left[n\right]$ is ${R}_{k}$, $\overline{{M }_{k}^{d}}$ denotes the dilated lesion mask with disk strel of radius d (a random value between 6 and 12). For the healthy samples, the constraint will be omitted. Only the two-branch network is adopted to classify the lesion category and segment the lesion area in the testing stage. We can get the predicted lesion category (DC, PC, AB, KCOT, and healthy) and the predicted lesion area mask for each input panoramic radiograph. Meanwhile, Grad-CAM^22^ can visualize the high response to the final predicted lesion category. The predicted lesion area mask and high response map visualized by Grad-CAM can be used as the diagnosis reference for the doctor to diagnose the cysts and tumors of the jaw.

## Results

### Lesion classification performance

**Figure 3:**
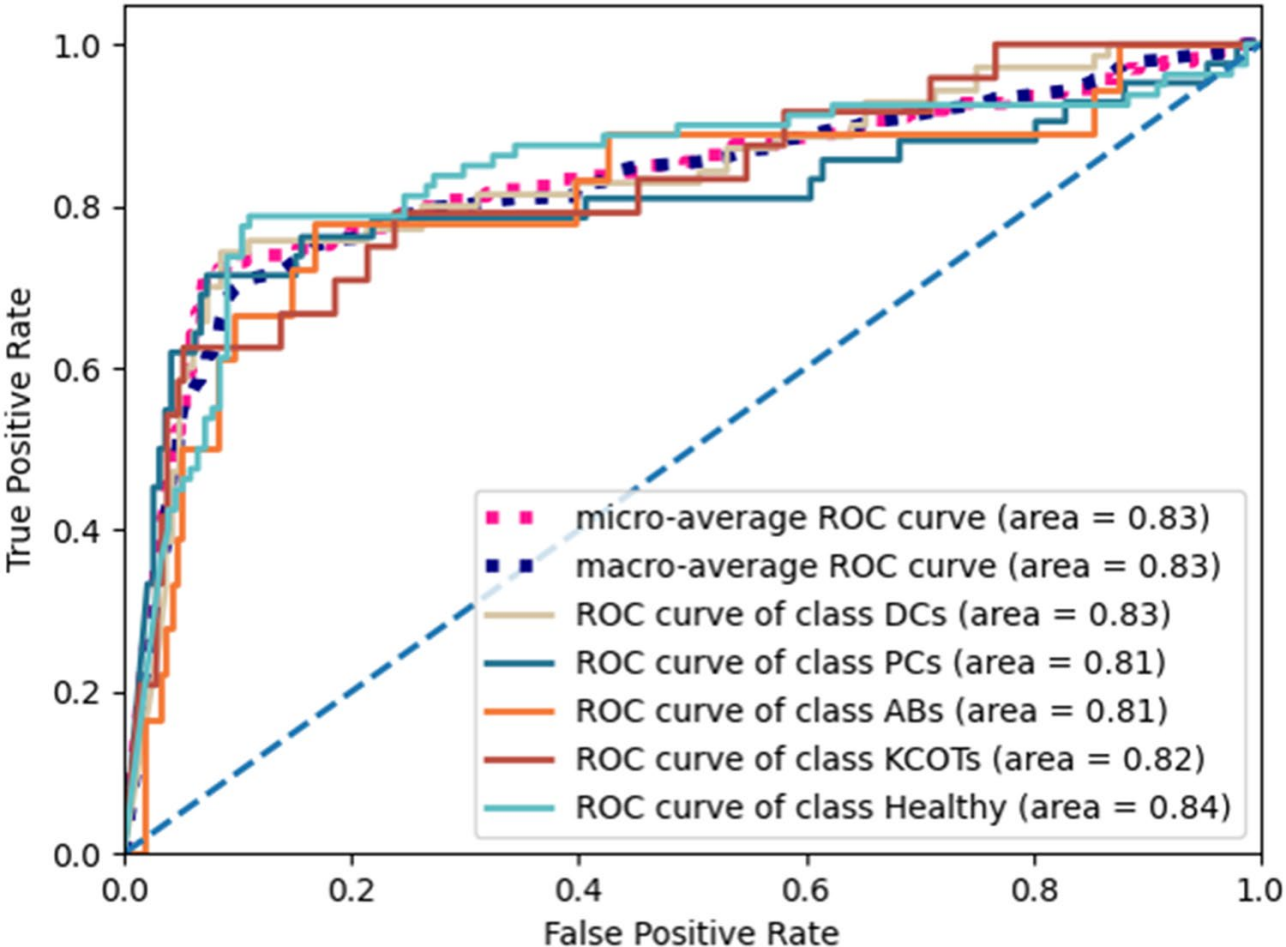
The ROCs and AUC scores of the five-class classification. Healthy samples achieve the highest AUC score than Cysts (DCs and PCs) and tumors (ABs and KCOTs).

In the experiment, the numbers of training, validation, and testing samples for each category are given in Fig. 1. The diagnosis of jaw cysts and tumors contains the binary classification and five-class classification. The five-class classification distinguishes the detailed category of the cyst, tumor, and healthy sample. Table 1 shows the five-class classification performance. The proposed method’s average accuracy, precision, sensitivity, specificity, and F1 score are 88.72%, 65.81%, 66.56%, 92.66%, and 66.14%, respectively. Our method achieves better classification performance than the existing three methods. Figure 3 shows the ROCs and AUC scores of the five-class classification, where we can see that the AUC scores of DCs, PCs, ABs, KCOTs, and healthy samples are 0.83, 0.81, 0.81, 0.82, and 0.84, respectively.

**Table 1:** The five-class classification performance of cysts, tumors and healthy samples.

	Category\index	Accuracy (%)	Precious (%)	Sensitivity (%)	Specificity (%)	F1-score (%)
Ours	DCs	86.32	78.79	74.29	91.46	76.47
	PCs	88.89	68.18	71.43	92.71	69.77
	ABs	91.45	45.00	50.00	94.91	47.37
	KCOTs	91.45	58.33	58.33	95.24	58.33
	Healthy	85.47	78.75	78.75	88.96	78.75
	Means	88.72	65.81	66.56	92.66	66.14
Ariji et al.^16^	DCs	72.03	53.85	49.30	81.82	51.47
	PCs	71.19	22.92	26.19	80.93	24.44
	ABs	85.17	18.52	27.78	89.91	22.22
	KCOTs	83.05	22.22	24.00	90.05	23.08
	Healthy	65.68	49.28	42.50	77.56	45.64
	Means	75.42	33.36	33.95	84.05	33.37
Kwon et al.^17^	DCs	69.79	49.09	38.57	83.03	43.20
	PCs	74.89	33.33	40.48	82.38	36.56
	ABs	85.11	16.00	22.22	90.32	18.60
	KCOTs	82.98	24.14	28.00	89.52	25.93
	Healthy	66.38	50.67	47.50	76.13	49.03
	Means	75.83	34.65	35.35	84.28	34.66
Yang et al.^26^	DCs	70.09	50.00	34.29	85.37	40.68
	PCs	74.36	32.00	38.10	82.29	34.78
	ABs	84.62	17.86	27.78	89.35	21.74
	KCOTs	80.77	13.79	16.67	88.10	15.09
	Healthy	63.68	46.84	46.25	72.73	46.54
	Means	74.70	32.10	32.62	83.57	31.77

Our method achieves higher scores than the other three methods on most metrics. For our method, all cysts and tumors have superior accuracy and specificity. Cysts (DCs and PCs) achieve higher sensitivity/recall scores than tumors (ABs and KCOTs).

**Figure 4:**
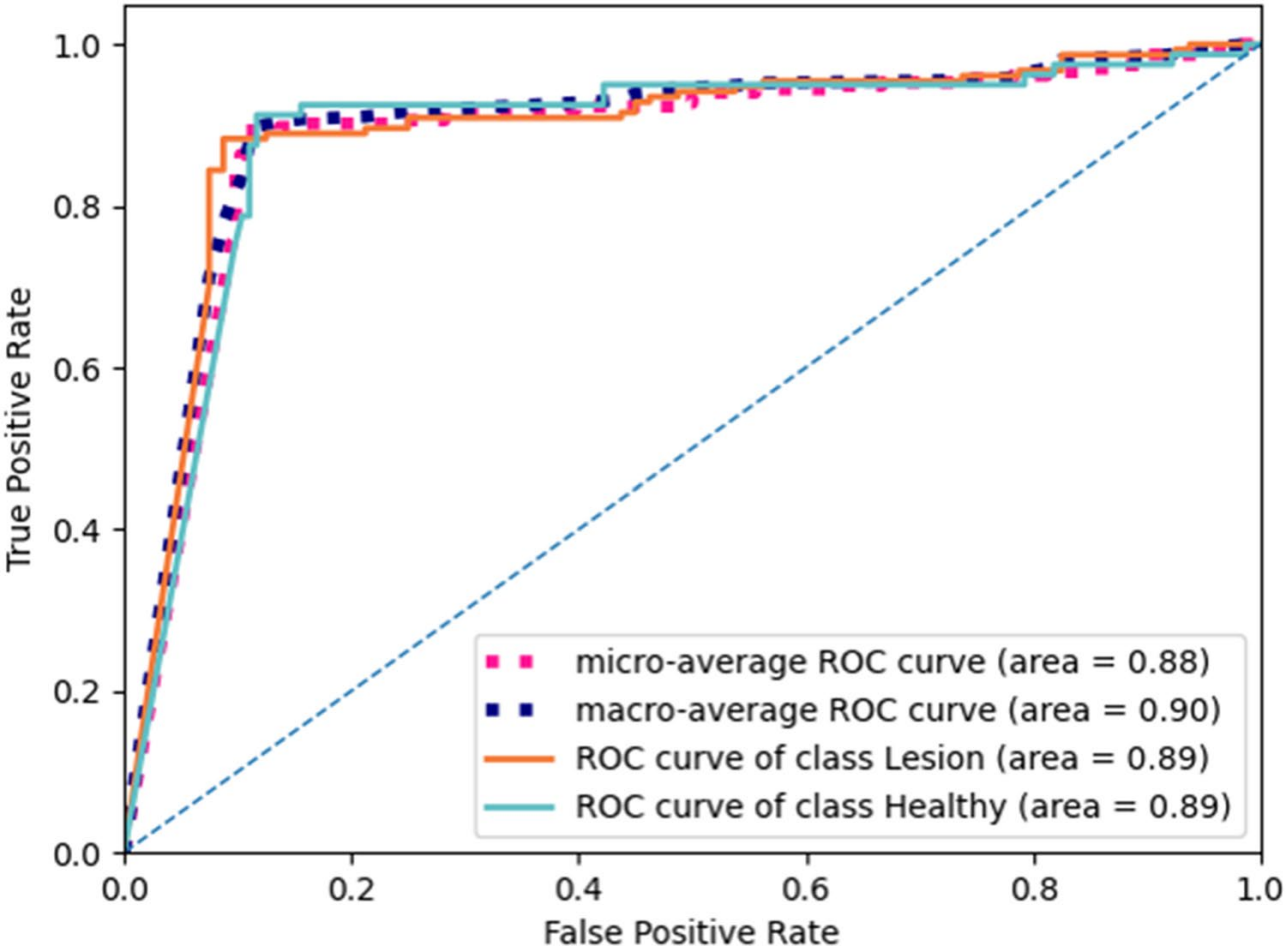
The ROCs and AUC scores of the binary classification. Lesion and healthy samples achieve the same AUC score.

Setting the ground-truth label as binary allows the two-branch network to be changed into the classifier for lesion and healthy samples. In the binary classification setting, the classification branch only classifies the lesion samples and healthy samples. Table 2 gives binary classification results of our method and other three works (Ariji et al.^16^, Kwon et al.^17^, and Yang et al.^26^). For our method, lesion and healthy samples both achieve 90.66% accuracy, which is higher than the average accuracy score of the five-class classification. Our method still achieves better binary classification performance than the existing three methods. Figure 4 shows the ROCs and AUC scores of the binary-class classification, where we can see that the AUC scores of the lesion and healthy samples are both 0.89.

**Table 2:** The binary classification performance of lesion and healthy samples.

	Category\index	Accuracy (%)	Precious (%)	Sensitivity (%)	Specificity (%)	F1-score (%)
Ours	Lesion	90.75	75.24	88.77	91.33	81.44
	Healthy	90.75	96.48	91.33	88.76	93.84
Ariji et al.^16^	Lesion	81.20	92.97	77.27	88.75	84.40
	Healthy	81.20	66.98	88.75	77.27	76.34
Kwon et al.^17^	Lesion	85.04	92.81	83.77	87.50	88.05
	Healthy	85.04	73.68	87.50	83.77	80.00
Yang et al.^26^	Lesion	82.05	93.08	78.57	88.75	85.21
	Healthy	82.05	68.27	88.75	78.57	77.17

Healthy samples achieve lower sensitivity/recall scores than lesion samples, which indicates that part of healthy samples tends to be classified as lesion samples. Our method achieves higher scores than the other three methods. The binary classification of our method achieves higher accuracy (90.66%) than the average accuracy score (88.72%) of the five-class classification in Table 1.

### Lesion area segmentation performance

Except for the classification performance, we give the segmentation performance of lesion samples. Furthermore, detection results of lesion areas are calculated by comparing the bounding boxes of predicted lesion masks between the ground-truth bounding boxes. Table 4 gives the segmentation and detection results of different lesion categories. For the binary classification and segmentation network, the segmentation and detection performance of lesion samples are given in Tables 5 and 6, where 85% of lesion samples can be detected by the proposed two-branch networks.

### Explainable results

**Figure 5:**
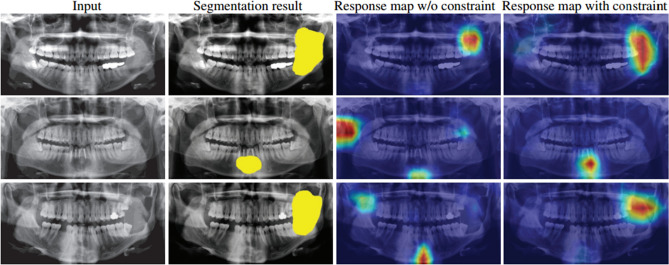
The segmentation results and visual results using Grad-CAM^29^. The segmentation results can locate the lesion areas. The response map can be used as the cause for explaining the classification results. For response maps w/o constraint, there are some inaccurate response areas, which will disturb the final classification and diagnosis. The response maps with the constraint have more concentrated response values than response maps w/o the constraint. With the accurate segmentation results and response maps as references, doctors can further confirm the diagnosis results.

Deep learning-based methods usually lack explainability, which is the primary drawback of deep learning-based methods. Medical image analysis requires that the predicted results are reliable and explainable. The proposed method can simultaneously predict the lesion category and area, increasing the reliability and explainability of the predicted results. Meanwhile, a position constraint is proposed to constrain the consistency between the segmented results and the response map of classification in the proposed method. Figure 5 gives the visual results of the original input, segmentation results, response map w/o the constraint, and response map with the constraint. The response map is visualized by the Grad-CAM^22^. For response maps w/o constraint, there are some inaccurate response areas, which will disturb the final classification and diagnosis. The response maps with the constraint have more concentrated response values than response maps w/o the constraint. There are some slight responses in the left part of the image in the first row and the last column. The contrast between the slight responses and the high responses is large. The constrain can reduce the disturbance of unrelated responses dramatically. With the accurate segmentation results and response maps as references, doctors can further confirm the diagnosis results.

## Discussion

From Table 1, we can see that all cysts and tumors have superior accuracy and specificity. What’s more, cysts (DCs and PCs) achieve higher sensitivity/recall scores than tumors (ABs and KCOTs), which means that tumors are more likely to be misclassified. From Tables 3 and 4, we can see that cysts (DCs and PCs) have better segmentation and detection performance than tumors (ABs and KCOTs), which means that cysts have easily identifiable features for the deep model. This is consistent with the classification performance in Table 1 that cysts (DCs and PCs) achieve higher sensitivity/recall scores than tumors (ABs and KCOTs).

**Table 3:** The segmentation performance of different cysts and tumors.

Category\index	Pixel accuracy (%)	Sensitivity (%)	Specificity (%)	Intersection over union (%)
DCs	71.32	73.27	71.42	73.26
PCs	68.43	67.51	72.53	72.34
ABs	67.25	51.35	68.90	67.54
KCOTs	65.42	64.22	69.71	70.23
Means	68.11	64.09	70.64	70.84

Cysts (DCs and PCs) have better segmentation performance than tumors (ABs and KCOTs).

**Table 4:** The detection performance of different cysts and tumors.

Category\index	Average precision (%)	Precious (%)	Sensitivity (%)	Intersection over union (%)
DCs	72.02	61.32	72.36	71.26
PCs	69.54	57.50	63.49	72.34
ABs	65.43	49.88	51.12	68.58
KCOTs	64.32	51.19	63.37	69.77
Means	67.83	54.97	62.59	70.49

Cysts (DCs and PCs) have better detection performance than tumors (ABs and KCOTs), which is consistent with the classification performance in Table 1 that cysts (DCs and PCs) achieve higher sensitivity/recall scores than tumors (ABs and KCOTs).

Tables 1 and 2 show that the binary classification achieves higher accuracy (90.66%) than the average accuracy score (88.72%) of the five-class classification, which means that the binary classification network is more suitable for distinguishing lesions from healthy samples. In Table 2, healthy samples achieve lower sensitivity/recall scores than lesion samples, which indicates that part of healthy samples tends to be classified as lesion samples. Furthermore, Tables 5 and 6 show that only distinguishing the lesion samples from the healthy samples can achieve more accurate segmentation and detection performance. Lesion samples have higher sensitivity/recall scores than healthy samples. For the healthy samples misclassified as lesion samples, the doctor can further verify the diagnosis results. This is an advantage of the binary classification network.

**Table 5:** The detection performance of lesion samples for the binary classification task.

Category\index	Average precision (%)	Precious (%)	Sensitivity (%)	Intersection over union (%)
Lesion	85.18	88.37	85.39	79.09

85% lesion areas can be detected by the proposed two-branch networks.

**Table 6:** The segmentation performance of lesion samples for the binary classification task.

Category\index	Pixel accuracy (%)	Sensitivity (%)	Specificity (%)	Intersection over union (%)
Lesion	82.56	84.62	81.79	73.94

Lesion samples achieves 82.56% pixel accuracy, which is consistent with the detection performance in Table 5.

In total, the binary classification achieves about 5–10% improvement than the five-class classification. We find that most misclassified lesion samples are classified into other kinds of lesions through statistics of misclassified samples. The tumors and cysts are easily misclassified, which is consistent with the clinical diagnosis. Odontogenic tumors and cysts do not reveal their distinct radiological characteristics until they reach a certain size. Early radiological appearances of odontogenic cysts and tumors are so indistinguishable from each other that even experienced oral and maxillofacial specialists are unable to guarantee their diagnosis results. In consideration of the better performance of the binary classification network, the results of the binary classification network can be used as the primary diagnostic reference. The predicted results of the five-class classification can be used for further diagnosis references. In clinical diagnosis, overall consideration of predicted binary and five-class classification networks results will achieve more reliable results.

Deep learning-based methods have achieved promising results in the medical image analysis area^7–9^. However, the deep learning-based methods have a severe deficiency that the inference process and predicted results are not unexplainable. Medical image analysis is a special scene that requires the diagnosis results have high reliability and explainability. For increasing the reliability and explainability, we add the segmentation branch in the proposed method. Meanwhile, the proposed position constraint, which constrains the consistency between the segmented results and the response map of classification, also improves the reliability and explainability of the predicted results. Figure 5 intuitively visualize the segmentation and response map results. The segmentation and response maps are an essential reference for the further diagnosis of doctors, which is the advantage of the proposed two-branch network. There are two factors for increasing reliability and explainability. Firstly, the segmented result of the lesion sample can be used as the diagnostic basis for the doctor to make further verification. Secondly, the patch-cover strategy is adopted to cover the random area of the lesion sample, which can increase the reliability of the prediction. For the lesion sample, the network should predict it as healthy if the lesion area is covered with a patch. On the contrary, the lesion sample is still predicted as a lesion if only the healthy area is covered with a patch. From Fig. 5, we can see that the lesion areas are accurately segmented. Table 7 shows that the classification performance will drop by about 5% accuracy without the segmentation branch, which indicates that the segmentation improves the explainability and the classification performance.

**Table 7:** The performance without pretrain and segmentation.

Category\index	Accuracy (%)	Precious (%)	Sensitivity (%)	Specificity (%)	F1-score (%)
-pretrain	75.33	34.45	35.67	84.48	34.22
-segment	84.35	54.23	59.01	89.78	53.32
whole	88.72	65.81	66.56	92.66	66.14

‘-pretrain’ and ‘-segment’ denote the network without the pretrain on healthy samples and segmentation branch, respectively. ‘whole’ denotes the whole framework.

Another deficiency of deep learning-based methods is that the performance of deep learning-based methods is very dependent on massive samples. However, collecting and annotating massive lesion samples is time-consuming and relies on the specialized knowledge of doctors. In this paper, we proposed a deep learning-based diagnosis method for cysts and tumors of the jaw with massive healthy samples. Table 7 gives the performance without pretrain on massive healthy samples, where we can see that the classification results will drop by about 13% accuracy without the pretrain on massive healthy samples. Like humans can learn prior knowledge from the normal samples, it is verified that deep learning-based methods can also learn some necessary knowledge from healthy samples. It’s an inspiration for further study on deep learning-based medical image analysis.

Overall, with massive healthy samples, the two-branch network achieves promising results for diagnosing cysts and tumors of the jaw. Except for the predicted categories, the two-branch network provides the segmentation results of lesion samples, which significantly improves the reliability and explainability of results predicted by deep learning-based methods. However, the proposed method can’t give the symptom causes why the lesion sample is classified as the specific lesion category, which is a significant research direction. In the future, we will focus on mining the symptom features of jaw cysts and tumors by adding attention mechanism.

## Conclusion

The cysts and tumors of the jawbone are usually painless and asymptomatic, which poses a serious threat to patient life quality. Proper and accurate detection at the early stage will effectively relieve patients’ pain and avoid radical segmentation surgery. Similar radiological characteristics of some cysts and tumors pose a severe challenge for the accurate diagnosis of cysts and tumors.

In this paper, we propose a deep learning-based method for diagnosing the cysts and tumors of the jaw. Unlike existing transfer learning-based methods, our proposed method can achieve promising diagnosis performance with massive healthy samples. We firstly collect 872 lesion panoramic radiographs and 10,000 healthy panoramic radiographs. Some data augmentation strategies are adopted to increase the diversity of training samples. Then, an encoder is pretrained on those massive healthy panoramic radiographs with self-supervised learning. Next, based on the pretrained encoder, a two-branch network is devised to classify the lesion category and segment the lesion area simultaneously. In the two-branch framework, the segmentation sub-network can effectively improve the classification performance and enhance the model’s explainability, which is advantageous for doctors to confirm the diagnosis result further. Further, the location consistency constraint is devised for constraining the consistency of predicted results between the segmentation sub-network and classification sub-network, which can effectively enhance the reliability and explainability of models. Exhaustive experiments demonstrate that the deep learning-based method achieves excellent results. The segmentation results can be served as reliable references for further diagnosis. It provides an effective tool for diagnosing cysts and tumors of the jaw. It’s worth noting that the proposed consistency constraint can be extended to other medical analysis areas, such as breast cancer analysis, hepatocellular carcinoma grading, brain diseases diagnosis. The predicted results with the consistency constraint are interpretable, which is more suitable for real medical diagnosis applications. The experiment results verify that the pretraining way effectively relieves the deep learning-based diagnosis method from relying on massive lesion samples, inspiring for future medical diagnosis tasks. Furthermore, we will focus on studying more techniques for improving the explainability of the medical diagnosis model in the future.

## Data Availability

The dental panoramic radiographs in the dataset used to develop the method and analyze the findings of this study are not publicly available due to the restriction by the First Affiliated Hospital, Zhejiang University School of Medicine in order to protect patients’ privacy.
